# Bulk segregant analysis coupled with transcriptomics and metabolomics revealed key regulators of bacterial leaf blight resistance in rice

**DOI:** 10.1186/s12870-023-04347-z

**Published:** 2023-06-22

**Authors:** Xiaozhi Ma, Manshan Zhu, Wuge Liu, Jinhua Li, Yilong Liao, Dilin Liu, Mengya Jin, Chongyun Fu, Feng Wang

**Affiliations:** 1 Rice Research Institute, Guangdong Academy of Agricultural Sciences, Guangzhou, China; 2 Guangdong Key Laboratory of New Technology in Rice Breeding, Guangzhou, China; 3 Guangdong Rice Engineering Laboratory, Guangzhou, China

**Keywords:** Bacterial leaf blight, Rice, Disease resistance, Genetics, R genes

## Abstract

**Background:**

Bacterial leaf blight (BLB) is a highly destructive disease, causing significant yield losses in rice (*Oryza sativa*). Genetic variation is contemplated as the most effective measure for inducing resistance in plants. The mutant line T1247 derived from R3550 (BLB susceptible) was highly resistant to BLB. Therefore, by utilizing this valuable source, we employed bulk segregant analysis (BSA) and transcriptome profiling to identify the genetic basis of BLB resistance in T1247.

**Results:**

The differential subtraction method in BSA identified a quantitative trait locus (QTL) on chromosome 11 spanning a 27-27.45 Mb region with 33 genes and 4 differentially expressed genes (DEGs). Four DEGs (P < 0.01) with three putative candidate genes, *OsR498G1120557200*, *OsR498G1120555700*, and *OsR498G1120563600,0.01* in the QTL region were identified with specific regulation as a response to BLB inoculation. Moreover, transcriptome profiling identified 37 *resistance analogs genes* displaying differential regulation.

**Conclusions:**

Our study provides a substantial addition to the available information regarding QTLs associated with BLB, and further functional verification of identified candidate genes can broaden the scope of understanding the BLB resistance mechanism in rice.

**Supplementary Information:**

The online version contains supplementary material available at 10.1186/s12870-023-04347-z.

## Background

Rice (*Oryza sativa*) is an important food security grain crop providing nutrition to 50% of the world population [1, 2]. There is an increasing demand with the increase in global population and adversities of climate change in terms of biotic and abiotic stresses [3]. Rice production is estimated to increase by up to 40% to meet the global demand by 2050 [4]. Increasing the yield of food crops is pertinent to meet the food security challenges. However, it is difficult to break the threshold due to domestication and adaptability bottlenecks in genetic improvement [5]. Therefore, reducing yield losses due to biotic and abiotic stresses is necessary. Biotic and abiotic stresses have significantly threatened crop production by decreasing crop yield [6, 7]. Breeding resilient cultivars by utilizing genetic sources is considered effective and eco-friendly for disease management [8].

Rice bacterial blight (BLB) is caused by *Xanthomonas oryzae* pv. oryzae [9]. BLB is a destructive bacterial disease in favorable tropical and temperate environments. Bacterial blight has a significant prevalence in southern China rice regions, threatening the safety of rice production [10, 11]. Due to the low control efficiency of chemical pesticides and the greater impact on the ecological environment, cultivating resistant varieties is the most economical and effective way to manage bacterial blight. The destructive nature of bacterial blight attracted many scientific studies, identifying key regulators in developing defense responses in rice, including biological and genetic control. Two classes of *R* genes exist in rice, receptor kinase (RLK) and NBS-LRR [12]. Until now, 47 *R* genes (*Xa1*-*Xa47*) have been identified in rice, and 8 have been physically mapped and cloned. Several studies have reported successful efforts with gene pyramiding resulting in durable resistance against BLB in rice [13–16]. With the onset of technological advancement, rapid identification of the genetic basis of disease resistance in plants is expedient.

In actual production, the pathogenic races continue to evolve with the growth of planting years. Hence, the resistance of some rice varieties introduced with bacterial blight resistance genes will be weakened or even lost. Therefore, it is pertinent to discover and identify new resistance genes. The use of molecular marker-assisted selection to breed new resistant varieties is of great significance for promoting high-yield, stable, and high-quality rice production. In this study, we utilized R3550 (BLB susceptible) and its mutant line T1274 (BLB resistant) to decipher the genetic background of disease resistance in T1274. Bulk segregant analysis (BSA), coupled with transcriptomics, was employed to further our understanding of the BLB resistance mechanism in rice.

## Methods

### Plant materials

In this study, two rice genotypes (*Oryza sativa*, subsp. indica L.), R3550 and T1247, were used. No permission is required to work on this species. Voucher specimens are available in the genebank herbarium of Guangdong Academy of Agricultural Sciences, Guangzhou, China, under XTP0973B67. Prof Chongyun Fu conducted the official identification of the plant material.

All plant materials were grown in paddy fields following normal field management practices. Each genotype was planted in blocks with four rows, and each row contained seven plants. At maturity, yield-related parameters, including the number of effective panicles, 500-grain weight (g), the number of grains, the number of empty grains, total grains, seed setting percentage, and the average number of grains per ear, were estimated following standard procedure. Morphological data was subjected to a *t*-test to estimate significant differences among genotypes.

### Bacterial culture and inoculation

At the adult stage, two genotypes, R3550, T1247, and their corresponding F2 population, were inoculated Xoo (*Xanthomonas oryzae* pv. oryzae). The lesions were investigated and sampled two weeks later. The inoculation was carried out by cutting leaf method, ten leaves were cut for each plant, and the average number of lesion lengths was taken. Thirty-six strains from the resistant and susceptible groups were mixed into a resistant pool, and a susceptible pool, respectively, and DNA was mixed to extract DNA for library building, sequencing, and analysis. The samples were further used for bulk segregant analysis (BSA) sequencing, transcriptome analysis, and targeted metabolomics.

### BSA sequencing

For BSA sequencing, fresh leaves from 72 F2 individuals (36 from resistant and susceptible pools each) along with two parents were collected from randomly selected plants. The genomic DNA was extracted, and individuals from resistant and susceptible groups (separately) were mixed in equal proportion and used for further analysis along with the parents. Libraries were constructed by sonicating the samples (M220 Covaris, Woburn, MA, USA) followed by polymerase chain reaction (PCR) amplification and quantification, and subjected to Illumina sequencing platform (Illumina, Inc., San Diego, CA, USA) for Hiseq X10 PE150 sequencing.

Sequencing data were aligned to the previously published genome Shuhui 498 (R498) (http://www.mbkbase.org/R498/) using BWA [17]. Before alignment, raw data were processed for quality control by removing reads with ≥ 10% unidentified nucleotides, Phred quality < 5, and not aligned > 10 reads. GATK pipeline was used for SNP calling [18]. The read-depth information for the single nucleotide polymorphism/ Insertion or deletion (SNP/InDel) index was estimated according to the method of Takagi et al. [19].

After filtering differential SNPs (870,275 SNPs) in both parents, their distribution of disease-resistant and susceptible pools was mapped. We used the differential subtraction method to perform a genome-wide scan with a sliding window of 2 Mb. Subsequently, we identified significant QTLs with (SNPindex > 0.5) associated with BLB resistance.

To further narrow down the QTL interval, we designed the primers according to the SNP sites in the QTL interval. We used 36 susceptible F2 populations as templates and performed a comparison with the susceptible parent R3550 after primer amplification. Individual plants were swapped, and genetic distance was calculated using the following formulas.

$$\begin{array}{c}{\rm{Genetic}}{\mkern 1mu} {\rm{distance}}{\mkern 1mu} {\rm{ = }}{\mkern 1mu} {\rm{number}}{\mkern 1mu} {\rm{of}}{\mkern 1mu} {\rm{exchanged}}{\mkern 1mu} \\{\rm{individual}}{\mkern 1mu} {\rm{plants}}{\mkern 1mu} {\rm{/}}{\mkern 1mu} \left( {{\rm{2*36}}} \right).\end{array}$$

The list of primers has been presented in Table S2.

### Transcriptome profiling

Transcriptome profiling was done using 12 libraries corresponding to randomly collected leaf samples (WT-0, MT-0, WT-1, and MT-1), each with three replicates. WT-0 and MT-0 represent the samples collected before inoculation, and WT-1 and MT_1 represent samples collected after inoculation. WT is R3550, while MT is T1247. After extraction of total RNAs with TRIzol reagent (TaKaRa, China), libraries (pair-end) were constructed using the Illumina HiSeq platform by the company Novogene (https://en.novogene.com/). We used FastQC for quality checks to remove low-quality reads and reads with < 50 bp sequence length [20]. FastQC is a widely used tool for the quality control of high-throughput sequencing data, including RNA-seq data. FastQC assesses the quality of reads based on several metrics, including per-base sequence quality, per-base sequence content, sequence length distribution, adapter content, and over-represented sequences. Moreover, we estimated Q20 and Q30 estimates as quality checks. Q20 and Q30 are quality scores that measure the percentage of bases with a sequencing error rate of less than 1% and 0.1%, respectively. Higher Q20 and Q30 scores indicate better sequencing quality and accuracy, meaning that a larger percentage of the reads will likely be correctly aligned to the reference genome or assembled de novo. We estimated GC contents for each sequencing library to ensure the quality of the data sets obtained. GC content is the percentage of guanine (G) and cytosine (C) nucleotides in the RNA-seq data. GC content is an important factor affecting the efficiency of library preparation, sequencing, and downstream analysis, such as alignment and quantification. Sequencing libraries with extreme GC content can cause amplification, sequencing, and alignment biases. Therefore, monitoring the GC content during quality control is important to ensure that the library is high quality and will produce reliable results.

Length, count and Fragments per kilobase per million mapped reads (FPKM) values for each gene were estimated and used for further downstream analysis. Differentially expressed genes (DEG) between different groups (WT-0, MT-0, WT-1, and MT-1) were analyzed using the DESeq R package (v1.18.0) [21]. Significant DEGs were screened using FDR ≤ 0.05 2-fold FPKM difference among samples. DEGs were annotated by utilizing the Gene Ontology (GO) and Kyoto Encyclopedia of Genes and Genomes (KEGG) databases [22].

## Results

### Morphological characterization

**Fig. 1:**
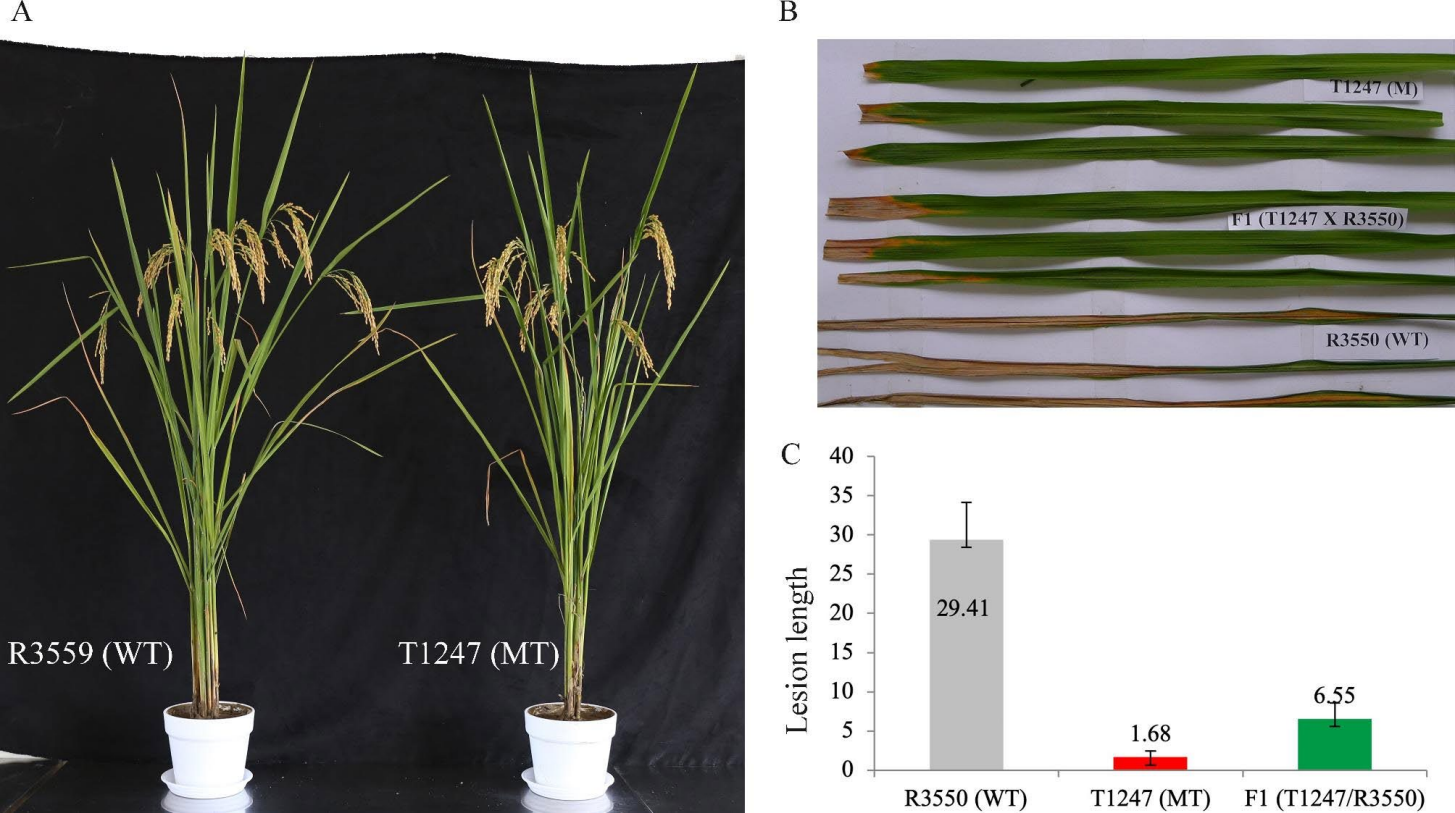
Phenotypic characterization of contrasting parents and F1 population. (**A**) pictorial description of R3550 (WT) and mutant line T1247 (MT) (**B**) Lesion length comparison of parental genotypes (R3550 and T1247) and their corresponding F1 population (**C**) bar plot for lesion length of parental genotypes (R3550 and T1247) and their corresponding F1 population

R3550 was treated with ethyl methanesulfonate (EMS)mutagenesis, and a gain-of-function dominant bacterial blight-resistant mutant T1247 was screened. After inoculation with bacterial blight, its lesion length significantly differed from R3550 (Fig. 1). However, overall, phenotypic characterization depicted a similar phenotype. Two contrasting genotypes were crossed to analyze the resistance inheritance, and the F1 genetic population was constructed. The F1 population was used for further characterization and analysis. Phenotypic characterization of bacterial leaf blight (BLB) depicted the prevalence of disease resistance against BLB (Fig. 1B). The average lesion length was considerably variable between parents and the F1 population. The average lesion length in susceptible parent R3550 was estimated as 29.41 cm, while it was 1.68 cm in the resistant mutant T1247. The F1 population depicted a slightly higher lesion length of 6.55 cm. The results signify the inheritance pattern of disease resistance from the mutant parent T1247.

**Fig. 2:**
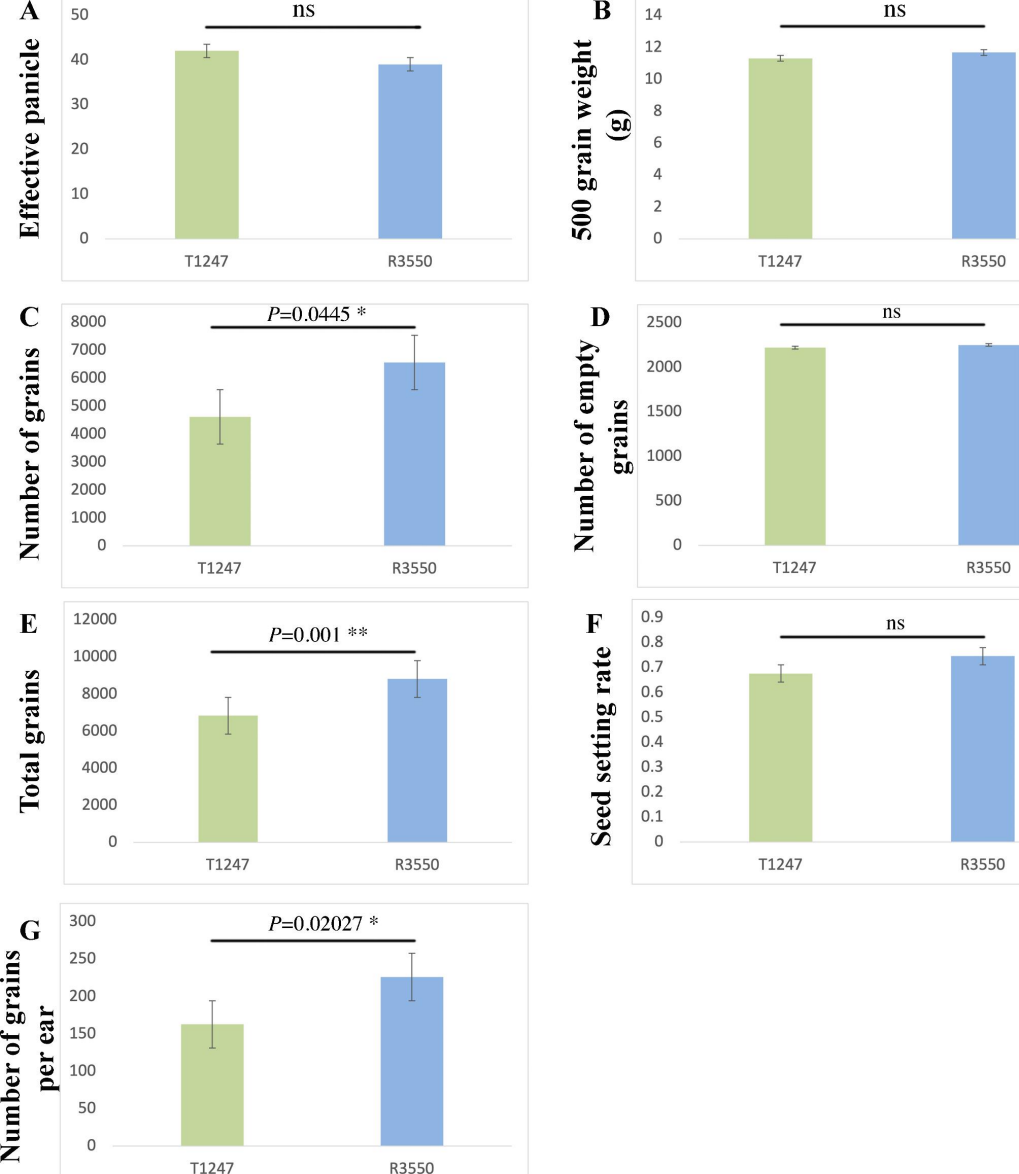
Estimation of yield-related traits in two contrasting genotypes R3550 and T1247. (**A**) number of effective panicles, (**B**) 500-grain weight (g), (**C**) number of grains, (**D**) number of empty grains, (**E**) total grains, (**F**) seed setting percentage, and (**G**) the average number of grains per ear

Moreover, we estimated the yield-related parameters, including the number of effective panicles, 500-grain weight (g), number of grains, number of empty grains, total grains, seed setting percentage, and average number of grains per ear. R3550 depicted a similar performance concerning all studied traits (Fig. 2).

### Bulk segregant analysis

**Fig. 3:**
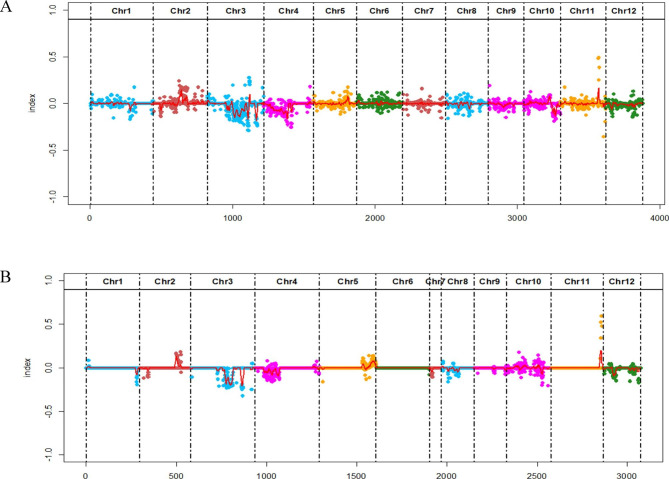
Bulk segregant analysis. (**A**) SNPs index T1247 (Mutant line) (**B**) SNP index R3550 (WT)

To explore and identify molecular markers associated with BLB disease in rice, SNP indices of each locus in parents, resistant pool, and susceptible pool were estimated using quality-filtered SNPs. The high-quality SNPs were classified as having a quality score ≥ 100 with a read depth ≥ 10. A total of 870,275 differential SNPs in parents were detected. Furthermore, SNPs distribution was estimated in the disease-resistant and susceptible pools. The differential subtraction method was used to perform a genome-wide sliding window analysis with a window of 2 Mb and a step size of 100 Kb. Subsequently, we identified a linkage interval located in chromosome 11 (Fig. 3), indicating the significance of this region’s association with BLB resistance. The QTL region (26,686,402–28,056,758 bp) was further narrowed down using SNPs-based markers (Table S1 and S2). The markers 2700-1 and 2745-1, with a genetic distance of 1.39 cM, narrowed down the QTL region to 27,000,022–27,452,291 bp. QTL region on chromosome 11, spanning 27-27.45 Mb region, consists of 33 genes (Table S3). The region was further characterized for gene regulation patterns with and without BLB inoculation in two genotypes.

### Transcriptome analysis

In order to identify key genes involved in the resistance to BLB, we further performed a transcriptome based on the RNA-seq approach. We constructed four libraries (WT-0, WT-1, MT-0, and MT-1), where WT-0 and MT-0 represent the samples collected before inoculation, and WT-1 and MT_1 represent samples collected after inoculation. The transcriptome profile of four samples was compared to comprehend the difference in expression profile associated with BLB resistance. A total of 113 Gb of data was generated. A total of 757,165,928 raw reads were obtained, and after filtering, 753,892,572 clean reads were kept (Table 1). The quality check was performed to confer the reliability and reproducibility of the data. Q20 and Q30 were estimated to be over 97 and 93%, respectively. Moreover, GC contents ranged from 49.42 to 52.58%. All the samples, including replicates, depicted significant correlation (Figure S1).

**Table 1:** Summary of transcriptome data generated from 12 libraries

Sample name	Raw reads	Clean reads	clean bases (G)	Error rate (%)	Q20(%)	Q30(%)	GC content (%)
MT_0_1	63,624,724	63,352,628	9.5	0.03	97.6	93.24	52.57
MT_0_2	61,189,646	60,958,612	9.14	0.03	97.87	93.82	51.26
MT_0_3	54,670,976	54,441,024	8.17	0.03	97.64	93.31	50.74
MT_1_1	45,619,778	45,425,488	6.81	0.03	97.69	93.41	49.42
MT_1_2	70,508,068	70,160,146	10.52	0.03	97.72	93.54	52.09
MT_1_3	68,403,978	68,091,982	10.21	0.03	97.63	93.34	51.57
WT_0_1	66,134,318	65,857,414	9.88	0.03	97.65	93.31	50.3
WT_0_2	67,724,378	67,441,994	10.12	0.03	97.77	93.61	50.15
WT_0_3	69,676,984	69,402,642	10.41	0.03	97.86	93.79	50.12
WT_1_1	73,875,964	73,532,390	11.03	0.03	97.55	93.17	51.8
WT_1_2	53,387,308	53,150,780	7.97	0.03	97.72	93.52	52.58
WT_1_3	62,349,806	62,077,472	9.31	0.03	97.67	93.43	51.09

**Fig. 4:**
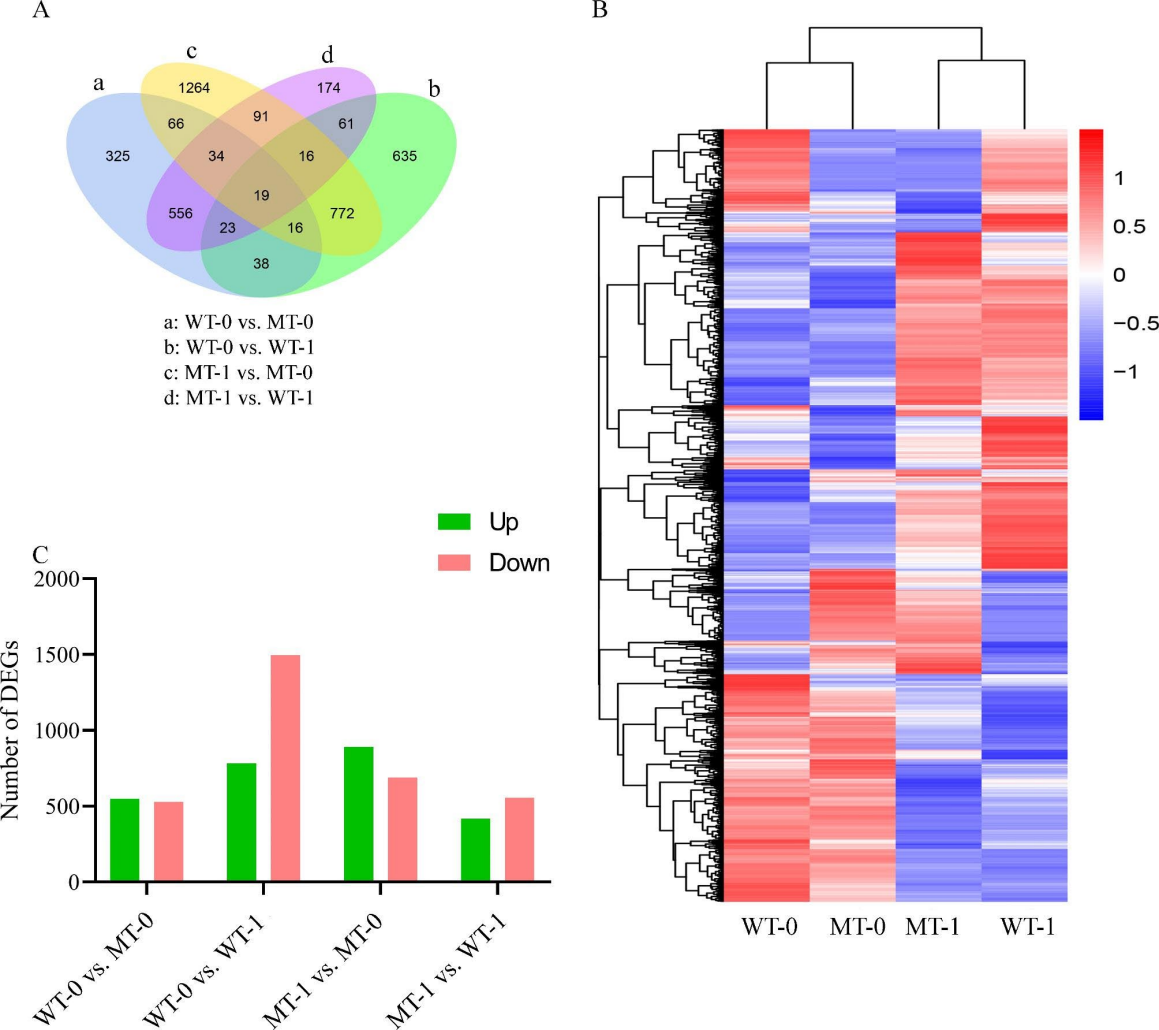
Transcriptome profiling. (**A**) Venn diagram representing overlapping DEGs between different groups (**B**) Heatmap depicting expression profiles of samples (WT-0, MT-0, WT-1, and MT-1) (**C**) number of differentially expressed genes in comparisons WT-0 vs. MT-0, WT-0 vs. WT-1, MT-1 vs. MT-0, and MT-1 vs. WT-1

Comparative transcriptome analysis between different groups identified 1078, 2278, 1580, and 974 differential expressed genes (DEGs) in comparisons WT-0 vs. MT-0 (550 upregulated and 528 downregulated), WT-0 vs. WT-1 (783 upregulated and 1495 downregulated), MT-1 vs. MT-0 (891 upregulated and 689 downregulated), and MT-1 vs. WT-1 (418 upregulated 556 downregulated), respectively (Tables S4, S5, S6, and S7). Moreover, comparisons WT-0 vs. MT-1 and WT-1 vs. MT-0 were identified with 1580 (891 upregulated and 689 downregulated) and 2380 (783 upregulated and 1595 downregulated) DEGs. We further identified 19 conserved DEGs with differential expression in all comparison groups in WT-0 vs. MT-0, WT-0 vs. WT-1, MT-1 vs. MT-0, and MT-1 vs. WT-1 (Fig. 4A C). The expression profile of all the DEGs identified has been presented in Fig. 4B, with evident differential regulation of these genes in different samples. The identified DEGs were further characterized for their associated GO and KEGG enrichment (Figures S2 and S3). Gene ontology analysis showed that the DEGs identified in different comparisons were significantly enriched in GO terms associated with plant disease responses, such as biosynthesis of steroids, lipids, secondary metabolites, etc. Moreover, KEGG enrichment also depicted a significant association of terms associated with disease-resistant responses.

### Transcription regulation of genes within QTL interval associated with BLB

The QTL region identified on chromosome 11 was further characterized using transcriptome datasets. Thirty-three genes were identified in the 45 Kb region (24-27.45 Mb). Twenty-three of these genes are known, while the remaining genes encode unknown proteins. Moreover, only 4 genes, including *OsR498G1120555700.01*, *OsR498G1120557200.01*, *OsR498G1120563600.01*, and *Novel00343*, were identified with differential expression (P < 0.01) in at least one comparison (Table S3). *OsR498G1120555700.01* encoding *PYRICULARIA ORYZAE RESISTANCE 21 (PI21)* was identified with up-regulation in comparison WT_0 vs. WT_1, while *OsR498G1120557200.01* encoding *LRR receptor-like serine/threonine-protein kinase* was identified with down-regulation expression in wild type (WT) under inoculation. *OsR498G1120563600.01* encoding *BTB/POZ and MATH domain-containing protein* showed differential expression patterns when wild-type and mutant lines were compared before and after inoculation (WT_0 vs. MT_0 and MT-1 vs. WT-1). In both genotypes, *OsR498G1120563600.01* depicted down-regulation after inoculation. The differential regulation pattern of these genes suggested BLB-specific resistance in response to BLB inoculation in both T1247 and R3550. The GO terms associated with identified genes in the QTL region emphasized the enrichment of GO terms associated with disease resistance, such as metabolic process, phosphorylation, oxidation-reduction process, and organic acid biosynthetic process (Table S3).

### Overall transcription response toward BLB resistance

Furthermore, to infer the genome-wide differential regulation of genes concerning BLB disease infestation, we compared R3550 and T1247 after inoculation. The results yielded differential regulation of 974 genes, with 418 upregulated and 556 downregulated genes (Table S6). The GO enrichment identified GO terms lipid biosynthetic process, oxidation-reduction process, steroid biosynthetic process, and steroid metabolic process. Further, we screened the known genes associated with BLB resistance in rice and identified 19 genes from the *resistance gene analogs (RGA)* family, including *RGA1*, *RGA2*, *RGA3*, *RGA4*, and their homologs. Among them, all the genes depicted higher expression in MT-1 (inoculated T1247) except five genes, including *OsR498G0100178200.01*, *OsR498G0100940200.01*, *OsR498G0202854900.01*, *OsR498G0511090900.01*, and *OsR498G0612961500.01* which were upregulated in WT-1 (inoculated R3550) (Table S6). The expression pattern of RGA genes suggested a significant role in developing resistance against BLB.

**Fig. 5:**
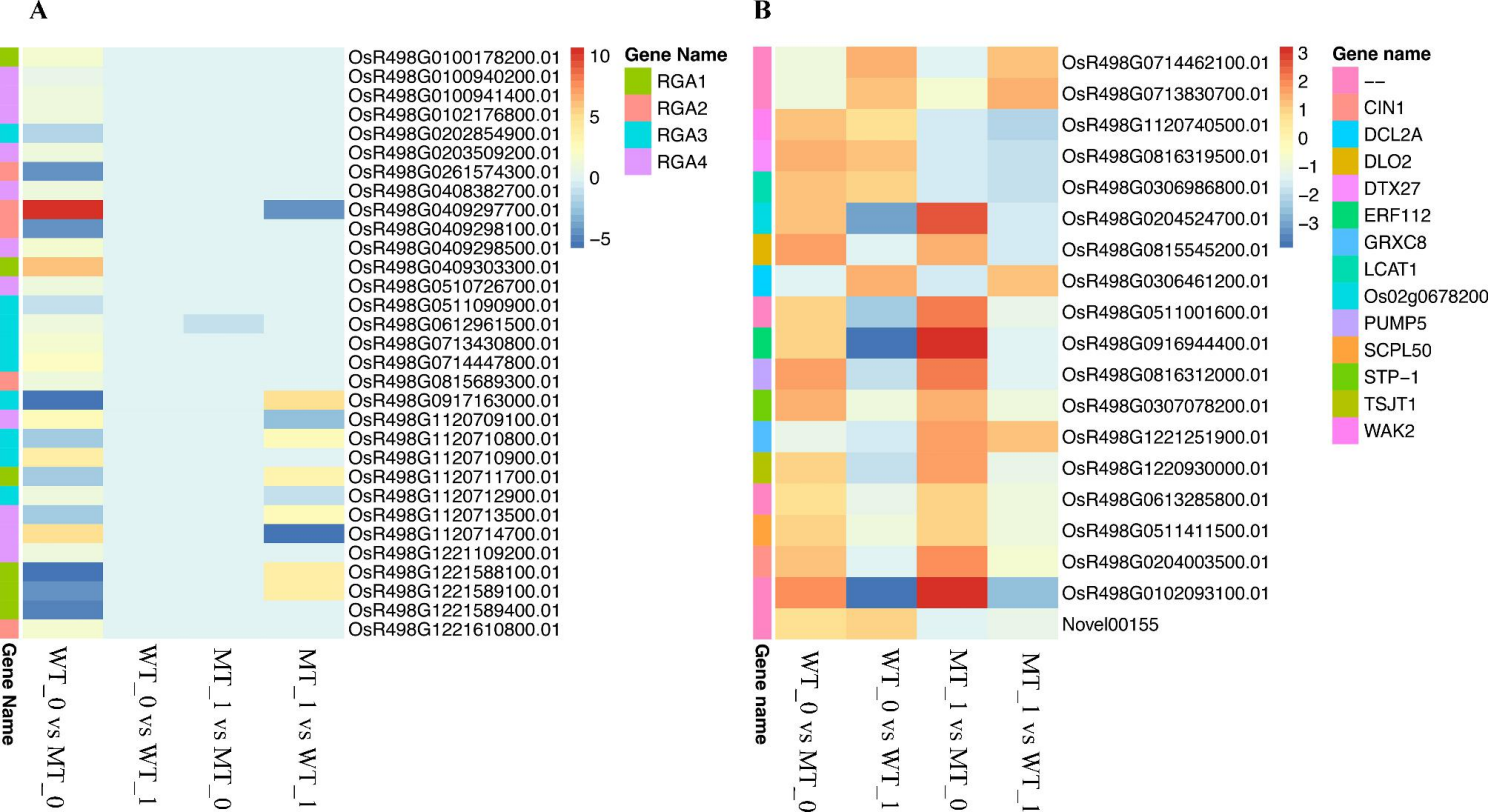
Disease responsive DEGs. (**A**) expression profile of 37 RGA family genes identified as differentially expressed in R3550 (WT) and T1247 (MT) with and without BLB inoculation (1/0). (**B**) expression profile of 19 conserved DEGs identified in different comparisons, including WT-0 vs. MT-0, WT-0 vs. WT-1, MT-1 vs. MT-0, and MT-1 vs. WT-1

Moreover, we identified 37 DEGs (WT-0 vs. MT-0, WT-0 vs. WT-1, MT-1 vs. MT-0, and MT-1 vs. WT-1) belonging to the *RGA* family and previously characterized as disease-resistance genes against BLB in rice (Table S4-S7). Most of the genes depicted differential expression patterns when compared without inoculation (WT-0 vs. MT-0), indicating that these disease-resistance genes have an active role in developing resistance against BLB in T1247 (mutant line-resistant). The results emphasized that the resistance mechanism in T127 is inherited, not acquired as a disease resistance response. Among the 31 DEGs, 11 were downregulated, and 20 were upregulated in WT-0 compared to MT-0 (Fig. 5A). Interestingly, when samples from both genotypes were compared with and without inoculation (WT-0 vs. WT-1 and MT-1 vs. MT-0), these *RGA* family genes showed no differential regulation. While after inoculation (MT-1 vs. WT-1) number of differentially expressed *RGA* genes were less than without inoculation (WT-0 vs. WT-0). RGA family genes differentially expressed in MT-1 vs. WT-1 and WT-0 vs. WT-0 comparison depicted opposite expression patterns in both comparisons, i.e., the upregulated genes in comparison WT-0 vs. WT-0 (without inoculation) were downregulated in MT-1 vs. WT-1 (inoculated). The results emphasized that disease incidence might have triggered these genes.

We further identified 19 genes with conserved differential expression in the four comparisons (Fig. 5B). The identified genes include *WAK2*, *DTX27*, *LCAT1*, *Os02g0678200*, *DLO2*, *DCL2A*, *ERF112*, *PUMP5*, *STP-1*, *GRXC8*, *TSJT1*, *SCPL50*, and *CIN1*. Most significant differences in expression patterns were observed when genotypes R3550 (WT) and T1247 (MT) were compared before and after inoculation. *Os02g0678200*, *DLO2*, *ERF112*, *PUMP5*, *STP-1*, *GRXC8*, *TSJT1*, *SCPL50*, and *CIN1* depicted downregulation in inoculated mutant line (MT-1) when compared before inoculation (MT-0), while their expression pattern was opposite in wild type (WT). Similarly, *WAK2*, *DTX27*, *LCAT1*, *DCL2A*, and *Novel00155* were upregulated in MT-0 compared with MT-1. Differential regulation of these genes under different treatments and comparisons suggested that these genes might have specific regulation under BLB incidence.

## Discussion

Bacterial leaf blight (BLB) is one of the most destructive diseases in rice, causing severe damage to the crop and ultimately reducing the yield. With the advancement of technology, several key genes have been identified with the positive control of BLB [23–28]. However, adaptive evolution in pathogens can produce resistance. Therefore, looking for new genetic resources for resistance development in plants is pertinent. For instance, Xa21, Xa23, Xa27, and Xa29 were identified in *O. longistaminata*, *O. rufipogon*, *O. minuta*, and *O. officinalis* and later introduced into cultivated rice and resulted in the development of several BLB resistant genotypes [23, 26, 29, 30]. In the current study, we systematically investigated R3550 (BLB susceptible restorer line) and its mutant T1247 (BLB resistant) by integrating bulk segregant analysis, transcriptome profiling, and targeted metabolomics.

We identified a QTL region on chromosome 11 (25-27.45 Mb) associated with BLB resistance in T1247. The region on chromosome 11 has been previously characterized in *Oryza rufipogon* [31] and they mapped a dominant resistant gene Xa47 at 26.24-kb on chromosome 11. 49 BLB resistance genes have been previously identified [2, 31]. We further explored the QTL region and identified 33 genes with 4 DEGs. *OsR498G1120557200.01* encoding (*R* gene) encoding *LRR receptor-like serine/threonine-protein kinase* was identified as DEG in the QTL region. However, it only depicted differential expression in the wild type (WT-0 vs. WT-1) and did not show a change in expression when the wild type and mutant genotypes were compared after inoculation. Our analysis of the BLB-resistant QTL revealed that while it is associated with BLB resistance, the expression pattern of the genes residing within this QTL did not significantly differ between the BLB susceptible line R3550 and its BLB-resistant mutant T1247. Therefore, it is possible that other mechanisms or factors (epigenetic modifications or post-transcriptional regulation) may be contributing to the resistance to BLB in T1247 rather than changes in gene expression within this QTL. Further investigation is needed to fully understand the underlying mechanisms of BLB resistance in T1247. Receptor kinase-like proteins are well known for their important role in strengthening the plant immune system and regulating growth and development [26, 32, 33]. The plant immune response involves cell surface-localized pattern recognition receptors (PRRs) and effector-triggered immunity (ETI) [11]. PRRs recognize pathogen-associated patterns and play a crucial role in the immune system. PRRs are either transmembrane receptor-like kinases (RLKs) or transmembrane receptor-like proteins (RLPs) [34]. Another important gene identified as DEG in the QTL region *OsR498G1120555700.01* encodes *PYRICULARIA ORYZAE RESISTANCE 21 (PI21)*. *PI21* belongs to the class of resistance genes, which has been extensively studied in rice against *Pyricularia oryzae* [11, 35–37].

BTB/POZ protein family is a key regulator in plant growth and development, with increasing evidence suggesting a significant role of the BTB/POZ protein family in plant defense regulation [38]. Our results depicted a down-regulated expression pattern of *OsR498G1120563600.01* encoding *BTB/POZ and MATH domain-containing protein* after BLB inoculation in both wild-type and mutant genotypes, suggesting specific downregulation in response to BLB inoculation. Zhao et al., studied the *BTB domain-containing protein* in tobacco and emphasized that *BTB* acts as a negative regulator of immunity response by suppressing effector-triggered HR [39]. Further functional characterization of *OsR498G1120563600.01* can provide significant insights into the regulation pattern on *BTB/POZ and MATH domain-containing protein* and its significance in developing BLB resistance in rice.

Moreover, we performed transcriptome profiling for two genotypes before and after inoculation to identify genome-wide DEGs. We identified several *resistance gene analogs (RGA)* genes, including *RGA1* (*Xa41*), *RGA2* (*XBQ99*), *RGA3* (*Xa40*), and *RGA4* (*Xa39*) and their homologs with differential expression patterns in at least one comparison. *RGA* genes have been characterized in multiple plant species for their active role in developing resistance against pathogens [2, 40–43]. For instance, Bayer et al., [44] 59 RGA candidates linked to Sclerotinia, clubroot, and Fusarium wilt resistance in *Brassica oleracea*. Moreover, *DLO2*, *ERF112*, *PUMP5*, *STP-1*, *GRXC8*, *TSJT1*, *SCPL50*, *CIN1*, *WAK2*, *DTX27*, *LCAT1*, *DCL2A* were identified with differential expression in each comparison WT-0 vs. MT-0, WT-0 vs. WT-1, MT-1 vs. MT-0 and MT-1 vs. WT-1. *DMR 6-LIKE OXYGENASE* [45], *ERF* [46], *PUMP* [47], *STP* [48], and *SCPL* [49] have been previously characterized for their potential role in inducing disease resistance responses in different plant species. Further functional insight into identified DEGs can provide exceptional input material for future breeding programs for BLB-resistant rice.

In brief, we systematically studied R3550 and its BLB-resistant mutant line T1247 to provide insight into BLB resistance and identified a QTL on chromosome 11 associated with BLB resistance. The transcriptomic profile identified several key genes associated with BLB resistance. Together, BSA and transcriptomics help mining of three putative candidate genes, *OsR498G1120557200*, *OsR498G1120555700*, and *OsR498G1120563600.01*,, for further study. Further insight into QTL and functional characterization of putative candidate genes can provide a valuable source for future breeding programs.

## Electronic supplementary material

Below is the link to the electronic supplementary material.

Supplementary Material 1

Supplementary Material 2

Supplementary Material 3

Supplementary Material 4

Supplementary Material 5

Supplementary Material 6

Supplementary Material 7

Supplementary Material 8

## Data Availability

The raw transcriptome data has been submitted to NCBI SRA under the accession number: PRJNA869771 (https://www.ncbi.nlm.nih.gov/bioproject/?term=PRJNA869771).
