# Supplementary material for: Bulk segregant analysis coupled with transcriptomics and metabolomics revealed key regulators of bacterial leaf blight resistance in rice
Source: BMC Plant Biol. 2023 Jun 22;23:332. doi: 10.1186/s12870-023-04347-z (PMC10286441; doi:10.1186/s12870-023-04347-z)
Supplement: Supplementary file 1 — Supplementary Material 1 [file 12870_2023_4347_MOESM1_ESM.docx]

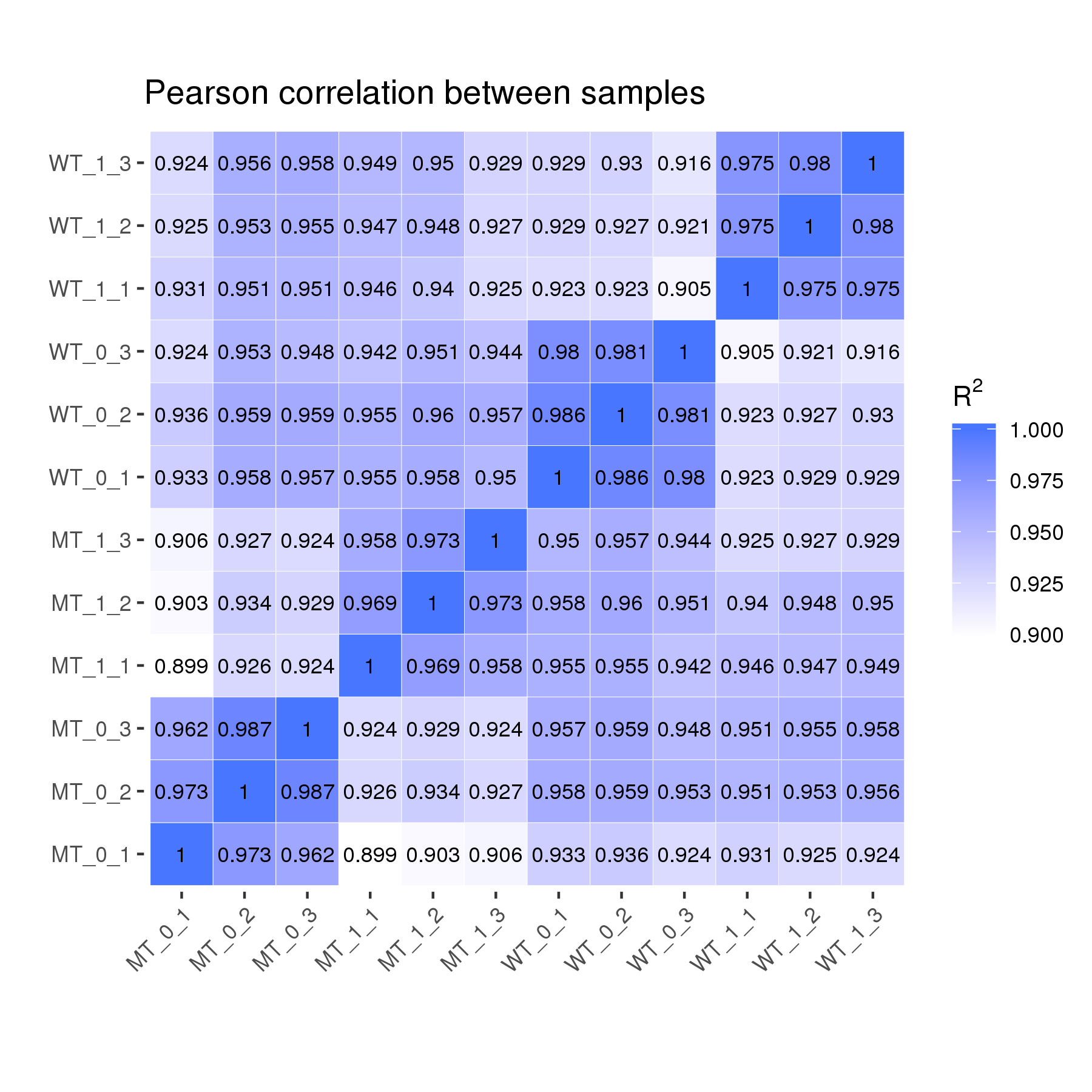
Figure S1. Pearson’s correlation of transcriptome datasets


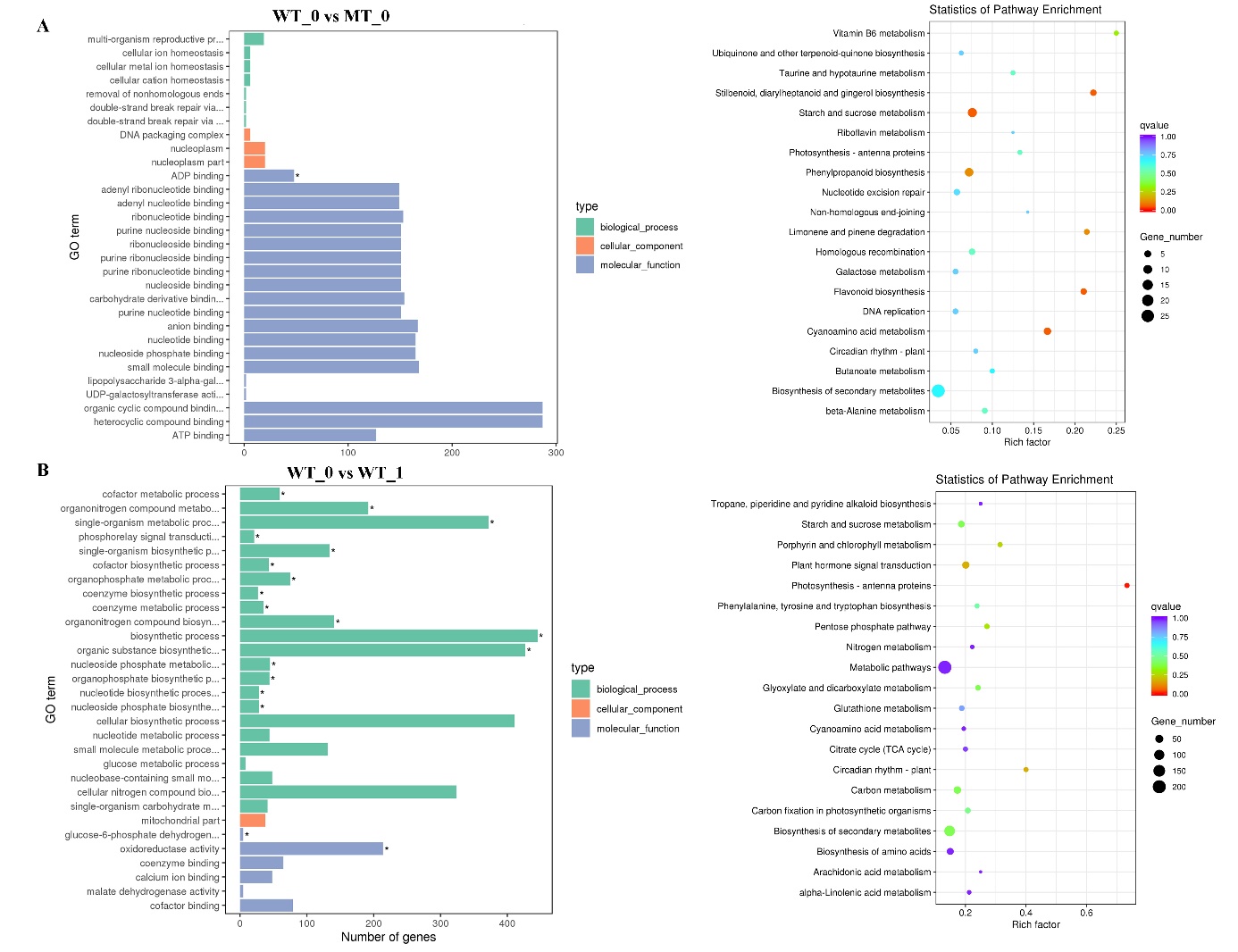
Figure S2. GO and KEGG enrichment for DEGs identified in WT_0 vs MT_0 and WT_0 vs WT_1


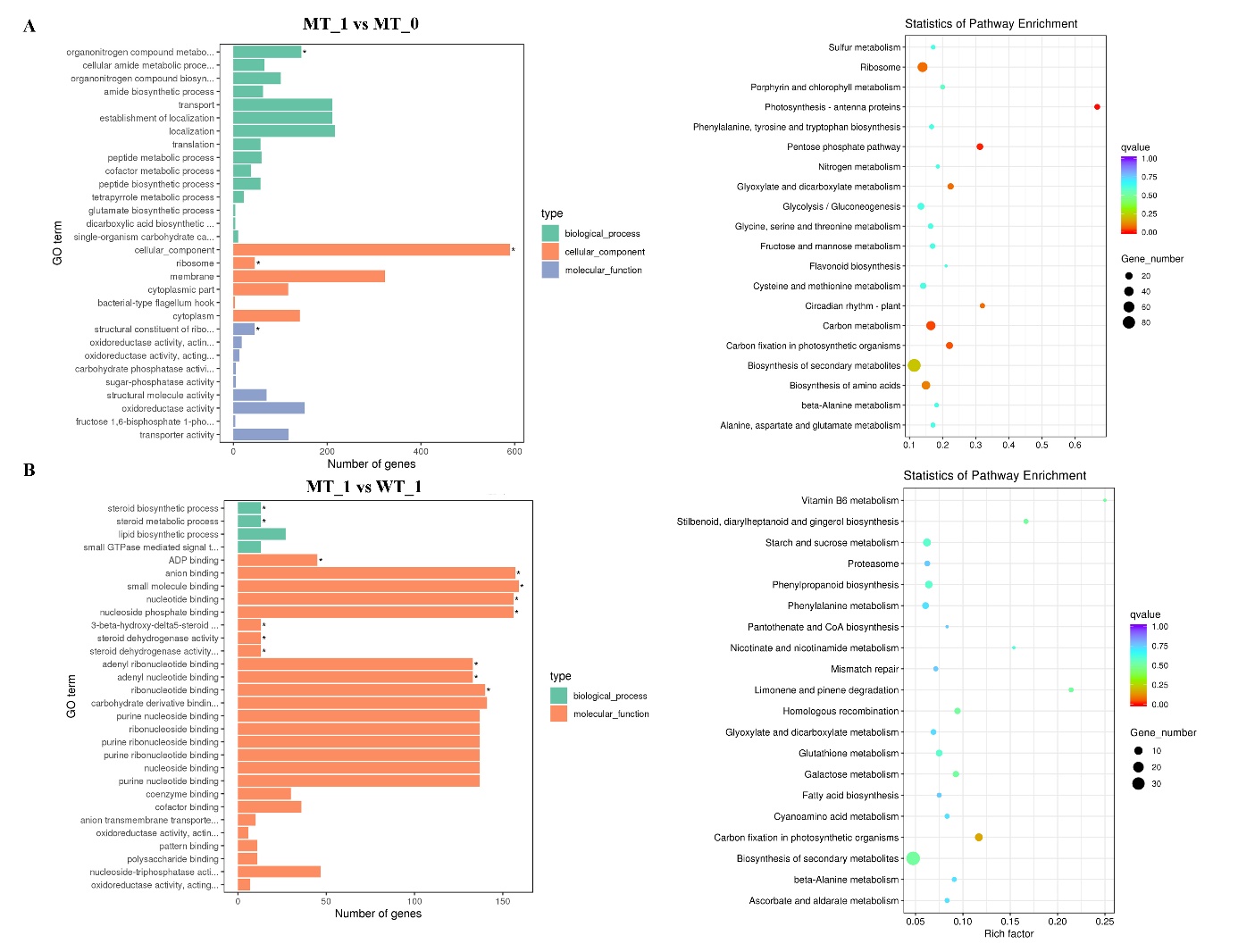
Figure S3. GO and KEGG enrichment for DEGs identified in MT_1 vs MT_0and MT_1 vs WT_1
